# STING-Pathway Inhibiting Nanoparticles (SPINs) as a Platform for Treatment of Inflammatory Diseases

**DOI:** 10.1021/acsabm.3c01305

**Published:** 2024-04-02

**Authors:** Lucinda E. Pastora, Neeraj S. Namburu, Karan Arora, Plamen P. Christov, John T. Wilson

**Affiliations:** † Department of Chemical and Biomolecular Engineering, Vanderbilt University, Nashville, Tennessee 37212, United States; ‡ School for Science and Math at Vanderbilt, Vanderbilt University, Nashville, Tennessee 37212, United States; § Vanderbilt Institute of Chemical Biology, Vanderbilt University, Nashville, Tennessee 37212, United States; ∥ Department of Biomedical Engineering, Vanderbilt University, Nashville, Tennessee 37212, United States; ⊥ Department of Pathology, Microbiology, and Immunology, Vanderbilt University Medical Center, Nashville, Tennessee 37232, United States; # Vanderbilt Institute of Nanoscale Science and Engineering, Vanderbilt University, Nashville, Tennessee 37212, United States; □ Vanderbilt Institute for Infection, Immunology, and Inflammation, Vanderbilt University, Nashville, Tennessee 37212, United States; ○ Vanderbilt Center for Immunobiology, Vanderbilt University Medical Center, Nashville Tennessee 37232, United States; △ Vanderbilt Ingram Cancer Center, Vanderbilt University Medical Center, Nashville, Tennessee 37232, United States; ▲ Vanderbilt Digestive Diseases Research Center, Vanderbilt University Medical Center, Nashville Tennessee 37232, United States

**Keywords:** STING, cGAS, inhibitor, nanoparticle, PLGA, controlled release, anti-inflammatory

## Abstract

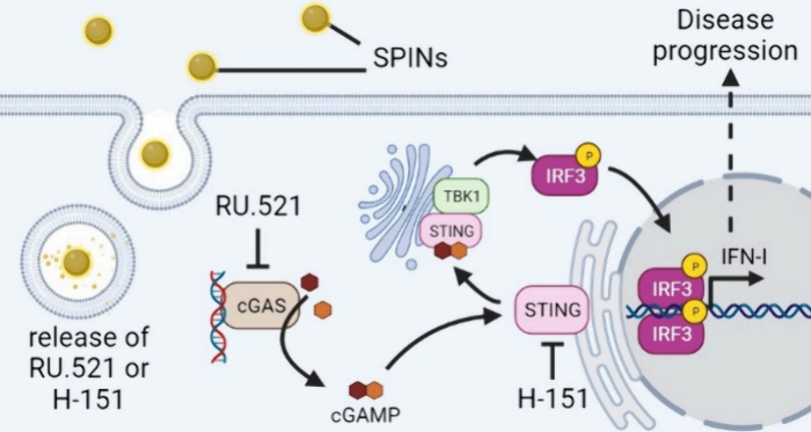

Aberrant activation of the cyclic GMP-AMP synthase (cGAS)/Stimulator of Interferon Genes (STING) pathway has been implicated in the development and progression of a myriad of inflammatory diseases including colitis, nonalcoholic steatohepatitis, amyotrophic lateral sclerosis (ALS), and age-related macular degeneration. Thus, STING pathway inhibitors could have therapeutic application in many of these inflammatory conditions. The cGAS inhibitor RU.521 and the STING inhibitor H-151 have shown promise as therapeutics in mouse models of colitis, ALS, and more. However, these agents require frequent high-dose intraperitoneal injections, which may limit translatability. Furthermore, long-term use of systemically administered cGAS/STING inhibitors may leave patients vulnerable to viral infections and cancer. Thus, localized or targeted inhibition of the cGAS/STING pathway may be an attractive, broadly applicable treatment for a variety of STING pathway-driven ailments. Here we describe STING-Pathway Inhibiting Nanoparticles (SPINS)–poly(lactic-*co*-glycolic acid) (PLGA) nanoparticles loaded with RU.521 and H-151–as a platform for enhanced and sustained inhibition of cGAS/STING signaling. We demonstrate that SPINs are equally or more effective at inhibiting type-I interferon responses induced by cytosolic DNA than free H-151 or RU.521. Additionally, we describe a SPIN formulation in which PLGA is coemulsified with poly(benzoyloxypropyl methacrylamide) (P(HPMA-Bz)), which significantly improves drug loading and allows for tunable release of H-151 over a period of days to over a week by varying P(HPMA-Bz) content. Finally, we find that all SPIN formulations were as potent or more potent in inhibiting cGAS/STING signaling in primary murine macrophages, resulting in decreased expression of inflammatory M1-like macrophage markers. Therefore, our study provides an *in vitro* proof-of-concept for nanoparticle delivery of STING pathway inhibitors and positions SPINs as a potential platform for slowing or reversing the onset or progression of cGAS/STING-driven inflammatory conditions.

## Introduction

The cyclic GMP-AMP synthase (cGAS)/Stimulator of Interferon Genes (STING) pathway plays a crucial part in the innate immune system, allowing cells to mount a pro-inflammatory response upon the detection of cytosolic double-stranded DNA.^1,2^ cGAS surveils the cytoplasm of cells, and upon recognition of double stranded DNA—often a sign of infection or cellular stress—catalyzes the production of 2,3-cGAMP, which in turn acts as a ligand for STING.^3^ cGAMP binds to STING located on the endoplasmic reticulum and initiates signaling that results in the phosphorylation and activation of Interferon Regulatory Factor 3 (IRF3) and the subsequent expression of type-I interferons (IFN-I) and pro-inflammatory cytokines.^4,5^ While this innate immune pathway is essential to healthy immune function, chronic or aberrant overactivation of the pathway can create an inflammatory environment that damages healthy tissue, can trigger cell death pathways, and can lead to organ pathology and even death.^6,7^ Genetic diseases such as Aicardi-Goutières syndrome (AGS) and STING-associated vasculopathy with onset in infancy (SAVI) are driven by mutations that result in constitutive, chronic activation of STING signaling,^8−10^ and a growing number of inflammatory diseases including ulcerative colitis,^11^ nonalcoholic fatty liver disease,^12,13^ and amyotrophic lateral sclerosis,^14^ have been linked to activation of cGAS/STING in response to cytosolic self-DNA induced by cell stress.

The importance of the cGAS/STING pathway in the onset and progression of such myriad inflammatory and autoimmune diseases has recently sparked interest in the development of pharmacological agents to block STING pathway activation, including small molecule competitive and covalent inhibitors and proteolysis-targeting chimeras (PROTACs),^15,16^ and plans have been announced to begin phase I clinical trials of a cGAS antagonist.^17^ Among the most promising agents reported to date are RU.521, a selective and potent cGAS inhibitor that occupies the active site of cGAS which decreases its affinity for ATP without affecting dsDNA binding,^18^ and H-151, a covalent STING inhibitor that blocks STING palmitoylation and subsequent interaction with TBK1, which is critical to STING signaling.^19^ Both of these inhibitors have improved outcomes in an expanding diversity of preclinical rodent models of diseases. RU.521 has been used to improve disease outcomes in rodent models of neuroinflammation caused by subarachnoid hemorrhage^20^ and cerebral venous sinus thrombosis,^21^ colitis,^22^ sepsis-induced organ injury,^23,24^ acetaminophen-induced liver injury,^25^ acute lung injury,^26^ hypertensive heart injury,^27^ ischemic injury in the lung^28^ and neonatal brain,^29^ femoral fracture healing,^30^ and aging-related endothelial dysfunction.^31^ Additionally, administration of H-151 has improved outcomes in rodent models of acute kidney injury (AKI),^32^ renal fibrosis,^33^ amyotrophic lateral sclerosis (ALS),^14^ sepsis-induced organ injury,^23,24^ psoriasis,^34^ ischemia-reperfusion injury in the intestine,^35^ myocardial infarction,^36^ LPS-induced acute lung injury,^37,38^ Alzheimer’s,^39^ and neuropathic pain resulting from chronic constriction injury.^40^ However, early experiences with these molecules has also identified several potential pharmacological barriers that may inhibit the translation of RU.521 and H-151 (and potentially cGAS/STING inhibitors more generally). Notably, nearly all previous studies leveraging these agents have used frequent, high-dose intraperitoneal injections, which is necessitated by the fast clearance of inhibitor from circulation (<2 h after intraperitoneal injection^19^). Not only would such frequent administration limit feasibility and translatability for most diseases, but systemic administration of cGAS/STING inhibitors may leave patients vulnerable to infection or cancer given the importance of the cGAS/STING pathway in antipathogenic defense^41−45^ and tumor immune surveillance.^46,47^ Additionally, while efforts are ongoing to develop orally bioavailable cGAS/STING inhibitors, currently both agents require solubilization in excipients such as corn oil^22^ or Tween-80 for administration.^36^ Thus, targeted inhibition of the cGAS/STING pathway in a relevant inflamed tissue or cell population may be an attractive, broadly applicable treatment for a variety of cGAS/STING-driven ailments, while avoiding the negative consequences of systemic STING inhibition.

Polymeric nanoparticles (PNPs) have been widely used to improve the stability and solubility of encapsulated small molecule drugs, target the drug to specific tissues or cell types, and increase drug circulation times to increase safety and efficacy.^48,49^ However, there exist very few reports describing PNPs for delivery of cGAS or STING inhibitors. Cheng et al. recently reported use of RU.521-loaded cationic PNPs delivered via subcutaneous hydrogel for rheumatoid arthritis immunotherapy,^50^ and, to our knowledge, no previous work has focused on PNPs for delivery of H-151. Here, we aimed to help fill this gap through the formulation and *in vitro* evaluation of STING pathway-inhibiting nanoparticles (SPINs), which are based on loading of H-151 and RU.521 into PLGA nanoparticles. We selected PLGA based on its high biocompatibility, tunable hydrolytic degradability into lactic acid and glycolic acid,^51^ and use in several FDA-approved products, including micro- and nanoparticles for delivery of small molecule immunomodulators.^52,53^ Based on their role in many cGAS/STING-driven diseases, we demonstrate the capacity of SPINs to inhibit cGAS and STING activation in macrophages, resulting in a reduction in IFN-I production and the prevention of STING-driven polarization toward an M1-like phenotype. SPINs therefore offer a modular platform for enhancing and sustaining inhibition of cGAS/STING signaling in inflammatory cell populations and establish proof-of-concept for the development of other STING-inhibiting nanoparticles formulations, which can ultimately be tailored to specific diseases, cellular targets, and/or route of administration.

## Results and Discussion

### SPIN-R Fabrication and *In Vitro* Characterization

**Figure 1:**
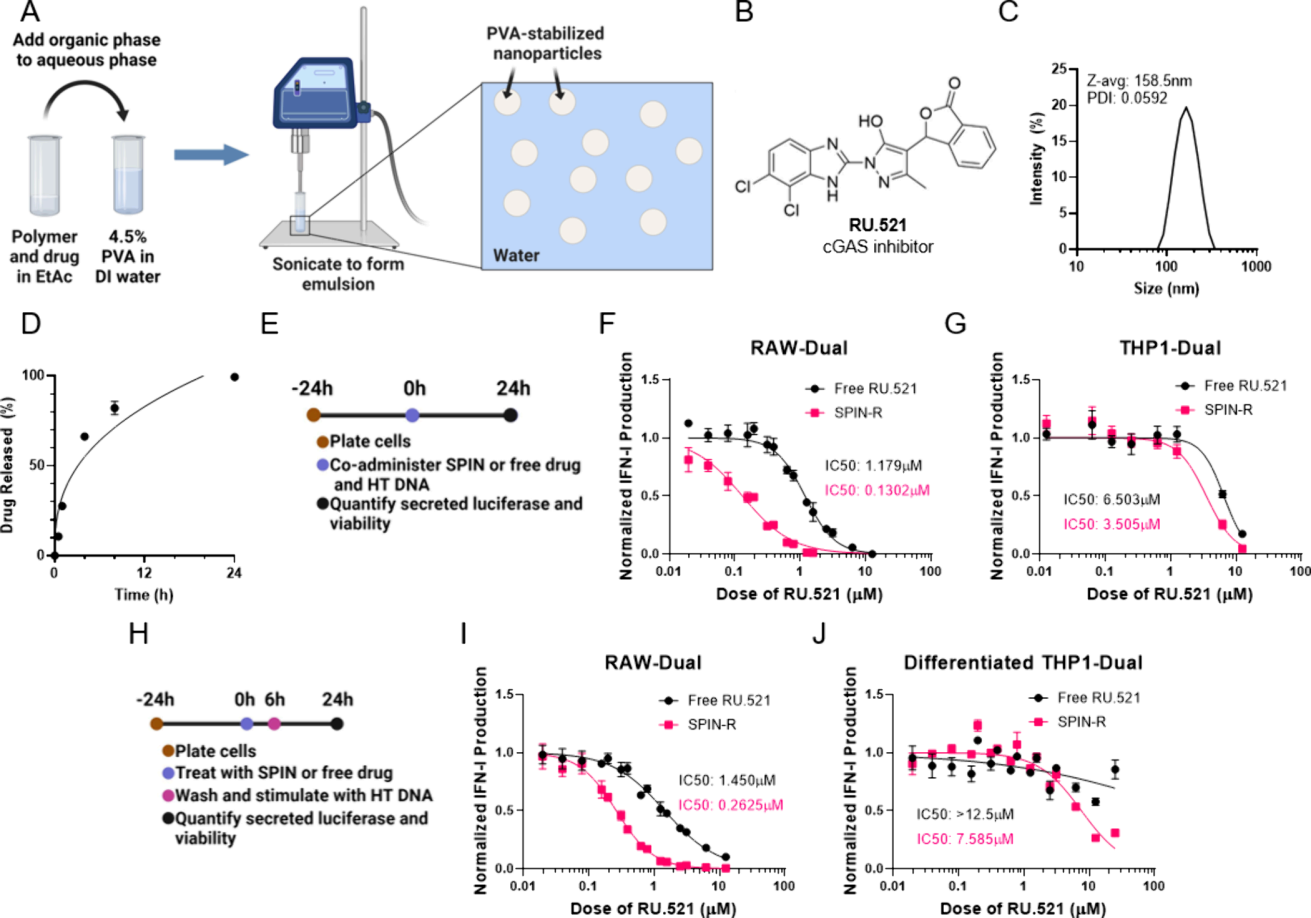
SPIN-R characterization and efficacy in cell lines. (A) Schematic depicting the oil-in-water fabrication method to produce SPINs. (B) RU.521 structure. (C) SPIN-R size distribution determined by dynamic light scattering. (D) RU.521 release from SPIN-R in pH 7.4 PBS at 37 °C. (E) Experimental timeline for testing SPIN-R and free RU.521 in reporter cell lines used in panels F and G. (F) Dose–response curves of IFN-I production induced by cotreatment with SPIN-R or RU.521 and 1 μg/mL HT DNA in RAW-Dual and (G) THP1-Dual cells (*n* = 3). (H) Experimental timeline for testing SPIN-R and free RU.521 in reporter cell lines used in panels I and J. (I) Dose–response of IFN-I production induced by pretreatment with SPIN-R or RU.521 prior to addition of 1 μg/mL HT DNA in RAW-Dual and (J) differentiated THP1-Dual cells (*n* = 3). Dose–response data is represented as mean ± SEM, fit using inhibitor vs response (four-parameter fit) nonlinear fit in GraphPad Prism.

To fabricate SPINs, we used an oil-in-water emulsion method (Figure 1A) to encapsulate the inhibitors into PLGA nanoparticles. Briefly, PLGA and the inhibitor of interest were dissolved in ethyl acetate and added dropwise to a solution of 4.5% PVA in DI water. The mixture was sonicated to form an emulsion, and ethyl acetate was evaporated on a rotary evaporator to harden the particles. The particles were then washed in DI water, flash frozen, and lyophilized to yield a dry stock that could be stored at −20 °C. We first fabricated RU.521-loaded SPINs (SPIN-R), which were 158.5 nm in diameter (Figure 1C) and contained 0.96% RU.521 by mass (Table 1).

**Table 1:** SPIN Formulation, Drug Loading, Size, and PDI

Formulation	Inhibitor	P(HPMA-Bz) excipient	Drug loading (%)	*Z*-average (nm)	PDI
**SPIN-R**	RU.521	none	0.96	158.5	0.0592
**SPIN-R**_**10%**_	RU.521	10 wt %	4.42	171.2	0.0674
**SPIN-H**	H-151	none	0.64	186.7	0.2573
**SPIN-H**_**1%**_	H-151	1 wt %	4.12	152.2	0.1178
**SPIN-H**_**5%**_	H-151	5 wt %	4.63	170.0	0.2229
**SPIN-H**_**10%**_	H-151	10 wt %	4.36	186.7	0.1159

We evaluated RU.521 release in PBS (pH 7.4) at 37 °C under constant orbital shaking, and found that SPIN-R fully released RU.521 within 24 h (Figure 1D). We then fit the release data to the first-order (eq 1), Baker–Lonsdale (eq 2), and Korsmeyer-Peppas (eq 3) models of drug release. For first order kinetics, release is dependent only the concentration of soluble agents in a porous matrix, and it is often used to describe release from a wide variety of drug delivery systems. The Baker–Lonsdale model is a modified version of the Higuchi model of drug release used for drug dispersed in matrices of spherical rather than planar geometries.^54^ Finally, the Korsmeyer-Peppas model is a semiempirical model that was developed specifically for drug release from polymeric systems of varying geometries, where the release exponent *n* provides information about mechanism of release. For spherical polymeric geometries, *n* = 0.43 for systems in which drug release is dominated by Fickian diffusion. However, *n* < 0.43 implies quasi-Fickian or hindered Fickian diffusion, while 0.43 < *n* < 0.85 is indicative of anomalous transport in which release is determined by other factors (such as polymer swelling or degradation) in addition to Fickian diffusion.^55^ As shown in Table 2, all three models appeared to be relatively good fits for SPIN-R, with first-order release best modeling the data. Additionally, the release exponent *n* = 0.374 indicates a quasi-Fickian release mechanism.

**Table 2:** SPIN Release Kinetics Parameters

	First-order		Baker–Lonsdale		Korsmeyer–Peppas		
**Formulation**	***k***	***R***^**2**^	***k***	***R***^**2**^	***k***	***R***^**2**^	***n***
**SPIN-R**	0.260	0.991	0.0192	0.975	0.328	0.932	0.374
**SPIN-R**_**10%**_	0.128	0.898	0.0137	0.967	0.210	0.894	0.492
**SPIN-H**	0.0576	0.520	0.00368	0.963	0.304	0.952	0.240
**SPIN-H**_**1%**_	0.0167	0.923	0.00171	0.946	0.0765	0.920	0.494
**SPIN-H**_**5%**_	0.0128	0.975	0.00143	0.964	0.0437	0.970	0.596
**SPIN-H**_**10%**_	0.00394	0.305	0.000219	0.929	0.0594	0.887	0.376

We next tested the activity of SPIN-R *in vitro* using RAW-Dual murine macrophage and THP1-Dual human monocyte reporter cell lines. Both cell lines stably express a Lucia luciferase gene that reports the activation of interferon regulatory factors, including IRF3. Thus, type-I interferon (IFN-I) production via IRF3 induction due to cGAS/STING activation can be monitored by measuring secreted Lucia luciferase activity on a luminescence plate reader. To stimulate the cGAS/STING pathway in these cells, we used double-stranded DNA isolated from herring testes (HT DNA), which is a commonly employed cGAS agonist that—when delivered cytosolically with a transfection agent such as Lipofectamine 2000—mimics a disease-relevant scenario of self-DNA sensing. SPIN-R or free RU.521 was codelivered with 1 μg/mL HT DNA to both RAW-Dual murine macrophages and THP1-Dual human monocytes (Figure 1E), and cGAS/STING activation was monitored 24 h later via supernatant luminescence. In both reporter cell lines, the SPIN-R more potently blocked cGAS/STING signaling compared to free RU.521, as demonstrated by the lower NP IC50 compared to free drug (Figures 1F and 1G). The IC50s of free RU.521 and SPIN-R were 1.179 μM and 0.1302 μM respectively in RAW-Dual cells, and 6.503 μM and 3.505 μM respectively in THP1-Dual cells. We also conducted pulse-chase studies in which cells were treated with SPIN-R for free RU.521 for 6 h, washed to remove noninternalized inhibitor, and then stimulated with HT DNA (Figure 1H). In this scenario, we again found that SPINs more potently inhibited cGAS/STING signaling than the free compounds. In RAW-Dual cells, IC50s of free RU.521 and SPIN-R were 1.450 μM and 0.2625 μM, respectively (Figure 1I), while the IC50s were >12.5 μM and 7.585 μM respectively in differentiated THP1-Dual cells (Figure 1J). We suspect that the increased benefit of SPINs in the pulse-chase study was due to the internalization of SPINS via phagocytosis or macropinocytosis within this 6 h incubation period. After the wash step, cells that had internalized the SPINS may have experienced the benefits of extended release of the inhibitors within cells, whereas free drug was washed away after 6 h. To further explore this suspected mechanism of action, we performed an uptake study in both RAW-Dual and THP1-Dual cells (Figure S1). Briefly, cells were treated with PLGA NPs loaded with Cyanine7 (Cy7) and incubated at 37 or 4 °C for 6 h. Then, cells were stained with DAPI, and the Cy7 signal in live single cells was determined using flow cytometry. Because cells cannot perform endocytosis or micropinocytosis at 4 °C, Cy7 signal in this group is a result of Cy7 release from the PLGA NPs; however, fluorescence in the 37 °C group is a result of both Cy7 release and NP uptake. Cy7 signal increased in both RAW-Dual and THP1-Dual cells, indicating that both cell lines are able to actively internalize our SPINs within a 6 h incubation period. Cell viability was high (≥70%) over the range of SPIN and RU.521 concentrations tested the dose–response studies described above (Figure S2). Interestingly, we also found that the highest concentrations of dose-matched empty PLGA NPs (>0.25 mg/mL) also inhibited the IFN-I response induced by HT-DNA in both macrophage lines tested, but not in undifferentiated THP1-Dual monocytes (Figure S3). This potentially implicates empty PLGA NPs as an indirect cGAS/STING inhibitor. Others have leveraged cargo-less PLGA NPs to inhibit TLR-driven inflammatory responses in macrophages,^56^ but further investigation is necessary for STING-driven inflammation. This anti-inflammatory effect may be, in part, due to the metabolic changes in the cells upon degradation of the PLGA and release of lactate.^57,58^

### SPIN-H Fabrication and *In Vitro* Characterization

**Figure 2:**
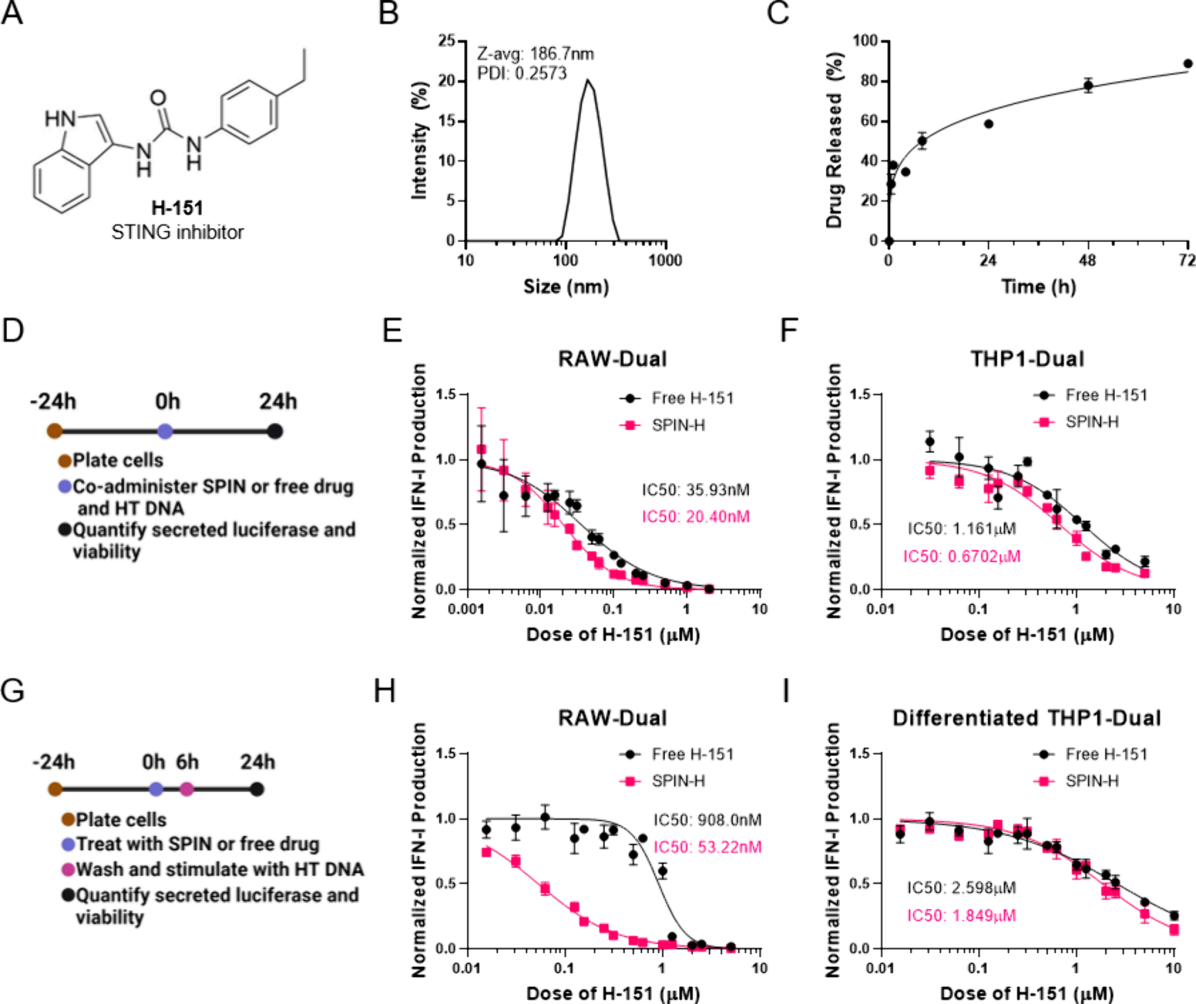
SPIN-H characterization and efficacy in cell lines. (A) H-151 structure. (B) SPIN-H size distribution determined by dynamic light scattering. (C) H-151 release from SPIN-H in pH 7.4 PBS at 37 °C. (D) Experimental timeline for testing SPIN-H and free H-151 in reporter cell lines used in panels E and F. (E, F) Dose–response curves of IFN-I production induced by cotreatment with SPIN-H or H-151 and 1 μg/mL HT DNA in (E) RAW-Dual and (F) THP1-Dual cells (*n* = 3). (G) Experimental timeline for testing SPIN-R and free RU.521 in reporter cell lines used in panels H and I. (H, I) Dose–response curves of IFN-I production induced by pretreatment with SPIN-H or H-151 followed by 1 μg/mL HT DNA in (H) RAW-Dual and (I) differentiated THP1-Dual cells (*n* = 3). Dose–response data is represented as mean ± SEM, fit using inhibitor vs response (four-parameter fit) nonlinear fit in GraphPad Prism.

We next fabricated H-151-loaded spins (SPIN-H) using the same oil-in-water emulsion method used for SPIN-R. This process yielded particles 186.7 nm in diameter as determined by dynamic light scattering (Figure 2B), and H-151 loading was 0.64 wt % in SPIN-H (Table 1). SPIN-H demonstrated a longer release profile than SPIN-R, and full H-151 release took about 3 days in PBS at 37 °C under constant orbital shaking (Figure 2C). We once again fit the release profile to the first-order, Baker–Lonsdale, and Korsmeyer–Peppas models of drug release (Table 2), and we found that the first-order model was not an appropriate model for H-151 release; instead, Baker–Lonsdale or Korsmeyer–Peppas were more appropriate models. Like SPIN-R, SPIN-H was fit with a release exponent *n* < 0.43, indicating quasi-Fickian diffusion from the PLGA nanoparticle matrix.

We next tested the *in vitro* activity of SPIN-H in RAW-Dual and THP1-Dual cells stimulated with HT DNA. Cells were cotreated with SPIN-H or free H-151 and 1 μg/mL HT DNA, and cGAS/STING activity was monitored 24 h later (Figure 2D). In both reporter cell lines, SPIN-H more potently blocked cGAS/STING signaling compared to free H-151. The IC50s of free H-151 and SPIN-H were 35.93 nM and 20.40 nM, respectively, in RAW-Dual cells (Figure 2E), and 1.161 μM and 0.6702 μM, respectively, in THP1-Dual cells (Figure 2F). In our pulse-chase studies (Figure 2G), SPIN-H exhibited enhanced benefit over free drug in RAW-Dual cells. This increased inhibitory activity, particularly in RAW-Duals was demonstrated to a greater extent than in the previous coincubation study. As has been observed using other NP-based formulations, this may reflect the generation of an intracellular drug depot that allows for sustained drug release and inhibition of cGAS/STING signaling. In the RAW-Dual cells, IC50s of free H-151 and SPIN-H were 908.0 nM and 53.22 nM respectively (Figure 2H), while IC50s were 2.598 μM and 1.849 μM respectively in differentiated THP1-Dual cells (Figure 2I). Macrophages are more endocytic than monocytes, which may contribute to the SPINs outcompeting free drug to a greater extent in RAW-Dual cells compared THP1-Dual cells. Furthermore, both of H-151 and RU.521 are more potent in mouse cells than human cells,^18,19^ which may also contribute to this observed effect. As with SPIN-R, cell viability was high (≳70%) over the range of concentrations tested (Figure S4).

Having tested SPINs in cell lines, we next sought to demonstrate their activity in primary murine macrophages. Bone marrow-derived macrophages (BMDMs) were cotreated with 2 μM SPIN-H or dose-matched controls and 1 μg/mL HT DNA. After 6 and 24 h, supernatants were collected for IFN-β ELISA and cells were lysed for RNA isolation and RT-qPCR analysis (Figure S5). After 6 h of treatment, SPIN-H significantly decreased IFN-β secretion compared to HT DNA alone, while free H-151 did not significantly decrease INF- β secretion (Figure S5A), but both treatments were significantly different from HT DNA alone by 24h (Figure S5B). Additionally, both free H-151 and SPIN-H decreased *Cxcl10* and *Ifnb1* expression compared to HT DNA treatment alone at 6 h (Figure S5C and S5D).

### Including an Excipient Polymer Increased Drug Loading and Extended Drug Release, While Maintaining SPIN Inhibitory Capacity

**Figure 3:**
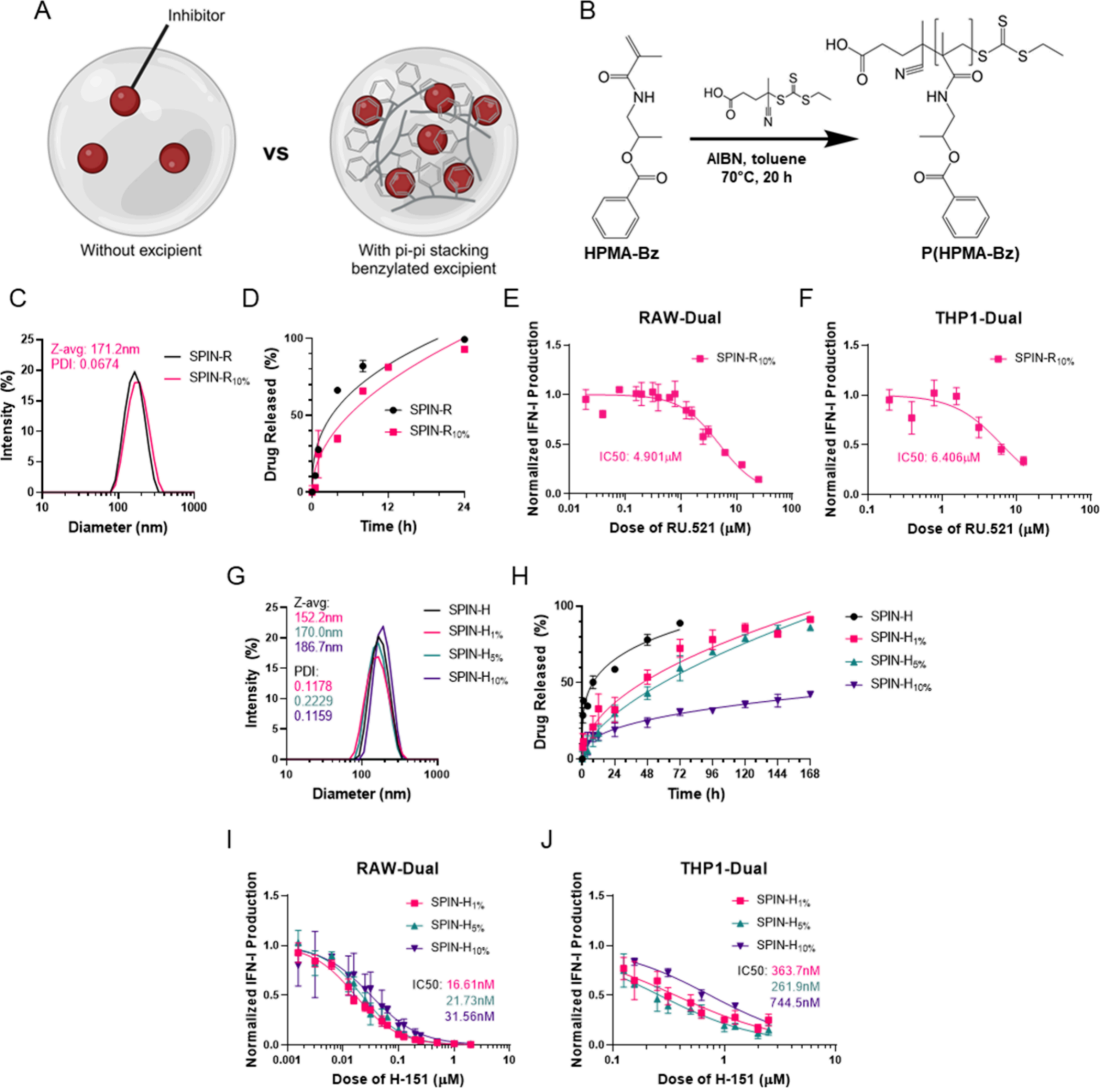
Including P(HPMA-Bz) in SPINs extends drug release without compromising inhibitor capacity. (A) Schematic of increased inhibitor loading with inclusion of benzylated excipient polymer in SPINs. (B) P(HPMA-Bz) synthesis scheme. (C) SPIN-R_10%_ size distribution determined by dynamic light scattering. (D) RU.521 release from SPIN-R_10%_ in pH 7.4 PBS at 37 °C. (E, F) Dose–response curves of IFN-I production induced by cotreatment with SPIN-R_10%_ and 1 μg/mL HT DNA in (E) RAW-Dual and (F) THP1-Dual cells (*n* = 3). (G) SPIN-H_1–10%_ size distribution determined by dynamic light scattering. (H) H-151 release from SPIN-H_1–10%_ in pH 7.4 PBS at 37 °C. (I, J) Dose–response curves IFN-I production induced by cotreatment with SPIN-H_1–10%_ and 1 μg/mL HT DNA in (I) RAW-Dual and (J) THP1-Dual cells (*n* = 3). Dose–response data are represented as mean ± SEM, fit using inhibitor vs response (four-parameter fit) nonlinear fit in GraphPad Prism.

Although SPINs were able to effectively inhibit the cGAS/STING pathway to a similar or greater capacity as respective free drug, both SPIN-R and SPIN-H nonetheless suffered from low drug loading (<1 wt %) and a relatively fast release profile that may limit applications and efficacy. We therefore explored the use of an excipient polymer to increase drug loading in our NPs. Inspired by the work of Ford et al.,^59^ we decided to blend in poly(benzoyloxypropyl methacrylamide) (P(HPMA-Bz)) to take advantage of pi-pi interactions between the excipient polymer and the inhibitors (Figure 3A). We synthesized P(HPMA-Bz) as shown in Figure 3B with a molecular weight of ∼20 kDa and a PDI of 1.05 (Figure S6). Then, to fabricate these second-generation SPINs, we again used the same oil-in-water emulsion method, but included 10 wt % P(HPMA-Bz) in the organic phase. RU.521-loaded PLGA nanoparticles containing 10 wt % P(HPMA-Bz) (SPIN-R_10%_) had an average diameter of 171.2 nm (Figure 3C), and drug loading increased more than 4-fold compared to SPIN-R (Table 1). While the addition of P(HPMA-Bz) improved loading of RU.521, drug release was not significantly affected as, like SPIN-R, SPIN-R_10%_ exhibited complete release of RU.521 within 24 h in 37 °C PBS (Figure 3D). Similar to SPIN-R, all three drug release models fit the experimental data well, but the release exponent *n* = 0.492 was characteristic of anomalous transport consisting of a mixture of Fickian diffusion and polymer swelling. Additionally, SPIN-R_10%_ inhibited cGAS/STING in RAW-Dual (Figure 3E) and THP1-Dual (Figure 3F) with slightly increased IC50s of 4.901 μM and 6.406 μM, respectively.

Next, we fabricated H-151-loaded PLGA nanoparticles containing 10 wt % P(HPMA-Bz) (SPIN-H_10%_), and drug loading improved approximately 7-fold from 0.64% w/w to 4.36% (Table 1) with little change to SPIN size (Figure 3G). Notably, SPIN-H_10%_ demonstrated extended drug release compared to excipient-free control, with less than half of the loaded drug being released over the course of a week (Figure 3H). The release profile was poorly fit using first-order release, but both the Baker–Lonsdale and Korsmeyer–Peppas models provided good fits to the experimental data. Additionally, and the release exponent *n* = 0.376 was characteristic of quasi-Fickian diffusion. Intrigued by this change in loading and drug release profile, we decided to vary the amount of excipient polymer used to formulate H-151-loaded nanoparticles. When we formulated SPINs with 5 wt % (SPIN-H_5%_) and 1 wt % (SPIN-H_1%_) excipient polymer, the resultant SPINs were similar in size to all previous formulations (Figure 3G), with average diameters of 150–190 nm. Additionally, SPIN-H_1%_ and SPIN-H_5%_ had similar loading as SPIN-H_10%_, which was ∼7-fold greater than SPIN-H drug loading (Table 1). H-151-loaded SPINs demonstrated tunable release, with increased P(HPMA-Bz) content correlating with longer drug release profiles (Figure 3H). SPIN-H_1%_ and SPIN-H_5%_ were well-fit by all three models of drug release, and both SPIN-H_1%_ and SPIN-H_5%_ had release exponent 0.43 < *n* < 0.85, indicating that both systems exhibit anomalous transport. Despite the slower drug release of these SPINS, the NPs demonstrated a similar capacity to inhibit cGAS/STING signaling caused by HT DNA treatment compared to SPIN-H in RAW-Dual (Figure 3I) and THP1-Dual cells (Figure 3J).

The release rates of the inhibitors are likely affected by many variables, including hydrophobicity of the inhibitor, ability to hydrogen bond and pi-pi stack, and potential interactions with the PVA stabilizer. RU.521 is slightly more hydrophilic than H-151, so it may diffuse more readily from the PLGA NPs, contributing to the faster release rate. Additionally, because of how quickly RU.521 is released, we suspect that RU.521 may associate with the PVA at the surface of the NPs. The inclusion of HPMA-Bz polymer slowed the release of both RU.521 and H-151 from SPINs, as demonstrated by the release profiles (Figure 3) and the release constants indicating anomalous transport (Table 2); however, this effect was likely amplified with H-151, as this inhibitor was slower to release at baseline, and likely experienced greater π–π interactions with HPMA-Bz compared to RU.521.

### SPINs Inhibit cGAS/STING Signaling in Primary Macrophages to Prevent a STING-Driven M1-like Phenotype

**Figure 4:**
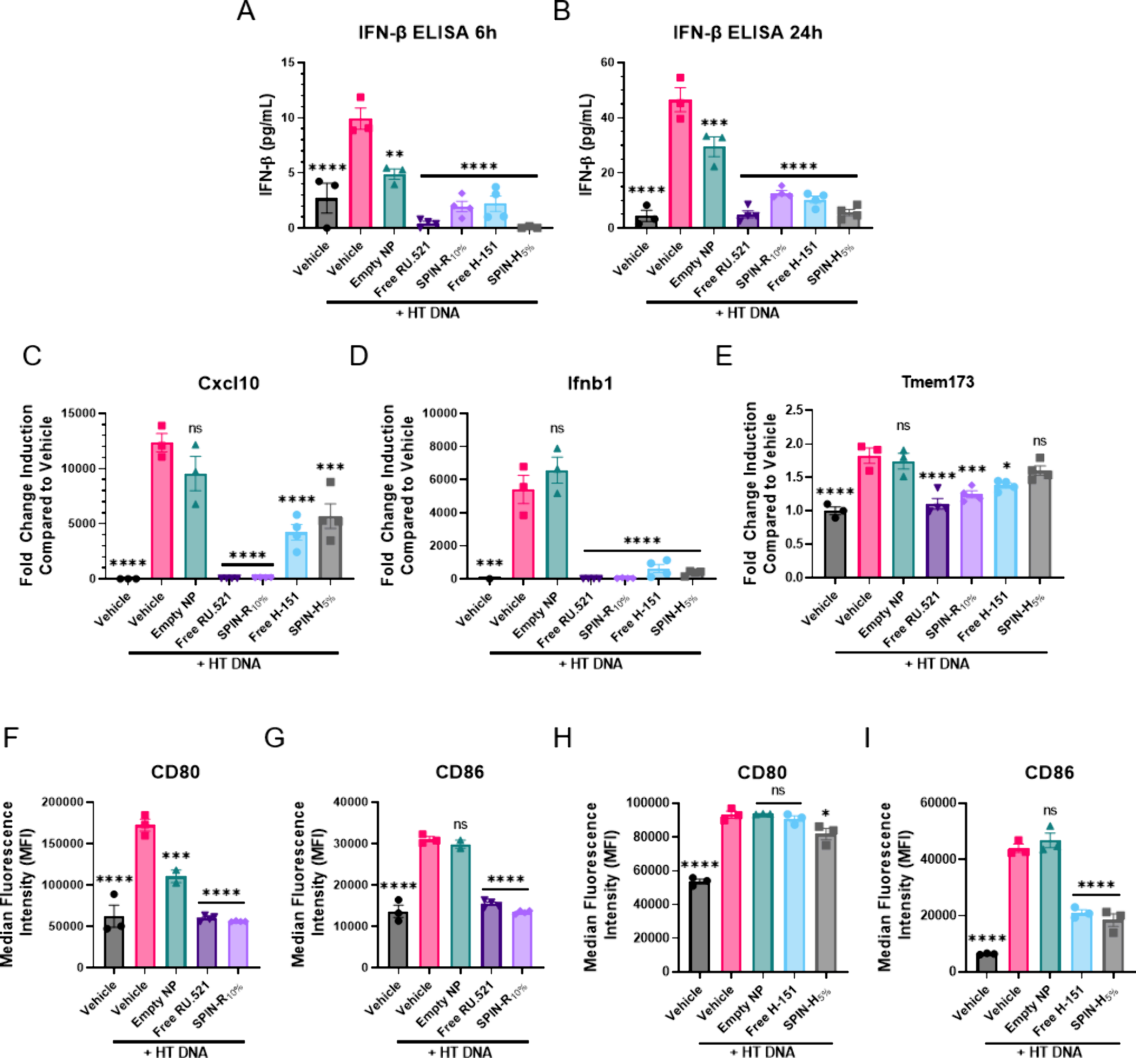
SPINs with P(HPMA-Bz) inhibit cGAS/STING signaling in BMDMs. (A, B) IFN-β ELISA of supernatants from BMDMs cotreated with the designated SPIN or control and HT DNA after (A) 6 h and (B) 24 h (*n* = 3–4). (C–E) Gene expression of (C) *Cxcl10*, (D) *Ifnb1*, and (E) *Tmem173* in BMDMs cotreated with the designated SPIN or control and HT DNA for 6 h (*n* = 3–4). (F, G) Surface expression of (F) CD80 and (G) CD86 in BMDMs cotreated with SPIN-RU_10%_, free RU.521, or empty NP and HT DNA for 24 h (*n* = 2–4). (H, I) Surface expression of (H) CD80 and (I) CD86 in BMDMs cotreated with SPIN-H_5%_, free H-151, or empty NP and HT DNA for 24 h (*n* = 3). Data are represented as mean ± SEM *P ≤ 0.05, **P ≤ 0.01, ***P ≤ 0.001, ****P ≤ 0.0001 compared to Vehicle + HT DNA by one-way ANOVA.

We screened excipient-containing NPs in primary cells, with a focus on SPIN-R_10%_ and SPIN-H_5%_, as this formulation demonstrated both increased drug loading and a moderate release profile. BMDMs were cotreated with 1 μg/mL HT DNA and 25 μM RU.521 in SPIN-R_10%_, 2 μM H-151 in SPIN-H_5%_, free inhibitor, dose-matched empty NPs, or vehicle. After 6 and 24 h, supernatants were collected for IFN-β ELISA and cells were lysed for RNA isolation and RT-qPCR analysis. The excipient-containing SPINs significantly decreased IFN-β secretion compared to HT DNA alone after 6 h (Figure 4A) and 24 h (Figure 4B) of treatment, demonstrating that the SPINs are able to inhibit cGAS/STING signaling to a similar capacity as free drug in primary macrophages. Additionally, SPIN-R_10%_, SPIN-H_5%_, and decreased *Cxcl10* (Figure 4C) and *Ifnb1* (Figure 4D) expression compared to HT DNA treatment alone. Interestingly, *Tmem173* (the gene encoding the STING protein) expression was significantly lower with SPIN-R_10%_ or free drug treatment, but not with SPIN-H_5%_ treatment (Figure 4E).

STING pathway activation in macrophages can promote polarization toward a pro-inflammatory M1-like phenotype^60,61^ with potential to drive disease pathology. For example, in inflammatory bowel diseases such as Crohn’s disease and ulcerative colitis, macrophages in patient mucosa predominantly shift to an M1 phenotype; these highly present macrophages are thought to drive intestinal inflammation and are associated with patient nonresponse to IBD treatment.^62−64^ Therefore, to test if SPINs could inhibit this M1-like polarization, we cotreated BMDMs with SPIN-RU_10%_, free RU.521, or empty NP and HT DNA. After 24 h, surface expression of CD80 and CD86 were measured via flow cytometry. Treatment with either SPIN-RU_10%_ or free RU.521 significantly decreased CD80 (Figure 4F) and CD86 (Figure 4G) expression compared to HT DNA treatment alone, and these cells were not significantly different from unstimulated BMDMs. Additionally, in BMDMs cotreated with SPIN-H_5%_, free H-151, or empty NP and HT DNA, only SPIN-H_5%_ treatment resulted in significantly lower CD80 expression on the surface of HT-DNA stimulated BMDMs (Figure 4H), while both SPIN-H_5%_ and free H-151 decreased CD86 expression compared to HT DNA alone (Figure 4I). Both CD80 and CD86 are costimulatory molecules that are highly expressed on activated pro-inflammatory M1 macrophages. Taken together with the ELISA and RT-qPCR data above, this implies that SPINs may decrease macrophage activation, which could be important to have a therapeutic effect in many STING-driven inflammatory diseases.

## Conclusions

In this work, we demonstrated the capacity of SPINs to inhibit cGAS/STING signaling in mouse and human macrophages and monocytes, thus reducing inflammation and inhibiting their polarization toward an M1-like phenotype. All nanoparticle formulations were as potent or more potent in inhibiting cGAS/STING signaling compared to dose-matched free drug in both human and murine macrophages, and treating BMDMs with SPINs decreased M1-like cell surface markers after stimulation with a cGAS agonist. Chronic, aberrant overactivation of the cGAS/STING has been implicated in a variety of inflammatory conditions, and the targeted delivery and sustained release of STING pathway inhibitors may help resolve inflammation and improve patient outcomes in STING-associated autoimmune and inflammatory diseases. This work offers a modular platform for enhancing and sustaining inhibition of cGAS/STING signaling in inflammatory cell populations and establishes a proof-of-concept for the development of other STING-inhibiting nanoparticles formulations, which can ultimately be tailored to specific diseases, cellular targets, and/or route of administration.

This technology may be particularly useful in situations in which cGAS/STING inhibition in a specific tissue is desired, and future work should be conducted to assess the therapeutic potential of SPINs in appropriate STING-driven inflammatory disease models. For example, SPINs may show promise as an intravenous therapy in cases of acute organ injury, such as acetaminophen-induced liver injury or sepsis-induced liver and kidney injury. In the case of acute or ischemic lung injury or AGS-induced lung injury, SPINs may be assessed as an intranasal therapy. SPINs may also have utility in neuroinflammatory conditions when delivered intrathecally, or in colitis when modified for oral or rectal delivery routes. With this system, we lay the groundwork for next-generating SPINs, which can be further designed with changes to size, geometry, targeting elements, etc., depending on the specific route of administration and desired release kinetics for assessment in a wide variety of STING-driven inflammatory conditions.

## Materials and Methods

### RU.521 Synthesis

#### Synthesis of 2,3-Dichloro-6-nitroaniline

In a heavy wall reaction vessel was added 1,2,3-trichloro-4-nitrobenzene (10 g, 0.044 mol) followed by ammonia in methanol (7 M, 100 mL). The reaction vessel was closed and heated at 100 °C for overnight. The solvent was removed in vacuum using rotary evaporator to give yellow solid that was used into the next step without purification. Yield 9 g (99%). ^1^H-NMR (d_6_-DMSO, 400 MHz) δ 8.10 (d, 1H, *J* = 12 Hz), 6.89 (d, 1H, *J* = 12 Hz); MS (ESI): mass calcd. for C_6_H_4_Cl_2_N_2_O_2_, 207.1; *m*/*z* found, 208.1 [M + H]+, *R*_f_ = 1.03.

#### Synthesis of 3,4-Dichlorobenzene-1,2-diamine

2,3-Dichloro-6-nitroaniline (9 g, 0.044 mol) was dissolved in methanol (200 mL). Added ammonium formate (3.04 g, 0.048 mol, dissolved in 10 mL of water) and slowly Zn (14.3 g, 0.22 mol, 5 equiv.). The reaction mixture was stirred at RT for 2 h. The solid was filtered off and the solvent was removed in vacuum. The solid was purified by Combi-flash, 0–100% hexane-ethyl acetate. Obtained 6 g (78%). ^1^H-NMR (d_6_-DMSO, 400 MHz) δ 6.81 (d, 1H, *J* = 12 Hz), 6.58 (d, 1H, *J* = 12 Hz), 3.90 (br s, 2H), 3.42 (br s, 2H); MS (ESI): mass calcd. for C_6_H_6_Cl_2_N_2_, 177.3; *m*/*z* found, 178.3 [M + H]+, *R*_f_ = 0.85.

#### Synthesis of 4,5-Dichloro-1,3-dihydro-2H-benzo[d]imidazol-2-one

3,4-Dichlorobenzene-1,2-diamine (6 g, 0.034 mol) was dissolved in anhydrous THF (100 mL). Added pyridine (5.5 mL, 0.068 mol, 2 equiv,) followed by dropwise addition of CDI (11 g, 0.068 mol, 2 equiv., dissolved in anhydrous DCM, 50 mL). The reaction mixture was stirred at RT for overnight. The reaction mixture was concentrated down to get a solid that was washed with hexane-ethyl acetate (10:1). Used in the next step without purification. Yield quantitative. ^1^H-NMR (d6-DMSO, 400 MHz) δ 6.81 (d, 1H, *J* = 12 Hz), 6.58 (d, 1H, *J* = 12 Hz), 3.90 (br s, 2H), 3.42 (br s, 2H); MS (ESI): mass calcd. for C_7_H_4_Cl_2_N_2_O, 203.1; *m*/*z* found, 204.1 [M + H]+, *R*_f_ = 0.95.

#### Synthesis of 2,6,7-Trichloro-1H-benzo[d]imidazole

4,5-Dichloro-1,3-dihydro-2H-benzo[d]imidazol-2-one (5.95 gr, 0.029 mol) was placed in a heavy wall vessel. POCl_3_ (100 mL) was added and the vessel was closed and heated at 120 °C overnight. The POCl_3_ was removed in vacuum and the residue was suspended in ice water. The solid was filtered off and washed with water to give white solid. Yield quantitative. ^1^H-NMR (d6-DMSO, 400 MHz) δ 7.62–7.49 (m, 1H), 7.43–7.45 (m, 1H); MS (ESI): mass calcd. for C_7_H_3_Cl_3_N_2_, 221.9; *m*/*z* found, 223.2 [M + H]+, *R*_f_ = 1.2.

#### Synthesis of 6,7-Dichloro-2-hydrazineyl-1H-benzo[d]imidazole

2,6,7-Trichloro-1H-benzo[d]imidazole (5 g, 0.023 mol) was added to a flask, followed by hydrazine hydrate (120 mL). The reaction mixture was heated at 100 °C overnight. The reaction mixture was cooled down and water (50 mL) was added. The solid was filtered off and washed with water. Obtained 4 g (80%). ^1^H-NMR (d_6_-DMSO, 400 MHz) δ 8.28 (s, 1H), 7.18 (brs, 1H), 7.06 (d, 1H, *J* = 8 Hz), 7.00 (d, 1H, *J* = 8 Hz), 4.70 (s, 2H); MS (ESI): mass calcd. for C_7_H_6_C_l_2N_4_, 217.0; *m*/*z* found, 219.0 [M + H]+, *R*_f_ = 0.85.

#### Synthesis of Ethyl 3-Oxo-2-(3-oxo-1,3-dihydroisobenzofuran-1-yl)butanoate

This compound was synthesized as previously described.^66^.

#### Synthesis of 3-(1-(6,7-Dichloro-1H-benzo[d]imidazol-2-yl)-5-hydroxy-3-methyl-1H-pyrazol-4-yl)isobenzofuran-1(3H)-one

In a dry flask, a suspension of 6,7-dichloro-2-hydrazineyl-1H-benzo[d]imidazole (1.74 g, 0.0081 mol) and 3-oxo-2-(3-oxo-1,3-dihydroisobenzofuran-1-yl)butanoate (2.33 g, 0.0089 mol) in glacial acetic acid (40 mL) was heated at 45 °C for overnight. The reaction mixture was poured into water and extracted with ethyl acetate (3 × 50 mL). The organic layers were washed with brine, dried with anhydrous MgSO_4_, and filtered off. Purified by Combi-flash Reverse Phase Column, 0–100% water (0.1% TFA)- acetonitrile. The appropriate fractions were combined, and solvent was concentrated in vacuum. The solid was filtered off and washed with water/methanol to give pure product as a mixture of enantiomers. Yield 2.83 g (85%). ^1^H-NMR (d_6_-DMSO, 400 MHz) δ 7.88 (d, 1H, *J* = 8 Hz), 7.77–7.74 (m, 1H), 7.62–7.57 (m, 2H), 7.44–4.42 (m, 1H), 7.35–732 (m, 1H), 6.61 (s, 1H), 2.19 (br s, 3H); MS (ESI): mass calcd. for C_19_H_12_Cl_2_N_4_O_3_, 414.3; *m*/*z* found, 415.3 [M + H]+, *R*_f_ = 0.99.

### H-151 Synthesis

In a dry flask, 3-isocyanato-1H-indole (1 gr, 0.0063 mol) and 4-ethylaniline (0.78 mL, 1 equiv., 0.0063 mol) were dissolved in anhydrous DMF (10 mL). The reaction mixture was stirred for 2h at RT. The reaction mixture was poured into water and extracted with ethyl acetate (3 × 50 mL). The organic layers were washed with brine, dried with anhydrous MgSO4 and filtered of. Purified by Combi-flash, 0–100% hexane-ethyl acetate. Obtained 1.5 g (85%) of white powder. ^1^H-NMR (d_6_-DMSO, 400 MHz) δ 10.68 (s, 1H), 8.46 (s, 1H), 8.37 (s, 1H), 7.50 (m, 2H), 7.48–7.31 (m, 3H), 7.10–7.06 (m, 3H), 7.01–6.97 (m, 1H), 2.55 (q, 2H, *J* = 8 Hz), 1.14 (tr, 3H, *J* = 8 Hz); MS (ESI): mass calcd. for C17H17N3O, 279.3; *m*/*z* found, 280.3 [M + H]+, *R*_f_ = 0.98.

### Benzoyloxypropyl Methacrylamide (HPMA-Bz) Monomer Synthesis

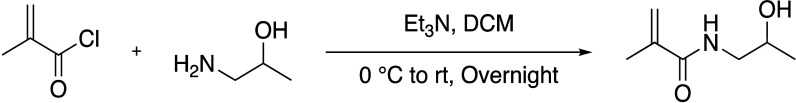



*N*-(2-hydroxypropyl)methacrylamide (HPMA) is created by starting with a solution of 1-aminopropan-2-ol (3.25 mL, 42 mmol, 1.1 equiv) and triethyl amine (6.4 mL, 46 mmol, 1.2 equiv) in 25 mL of dichloromethane maintained at 0 °C under a nitrogen atmosphere. A solution of methacryloyl chloride (3.74 mL, 38 mmol, 1.0 equiv) in 15 mL of dichloromethane was added dropwise to the reaction mixture with continuous stirring under an inert atmosphere. The reaction mixture was stirred at room temperature overnight. After consumption of the starting material, as judged by TLC analysis, the reaction mixture was filtered through Celite and the solvent was evaporated to get the crude product, which was purified by silica gel column chromatography using a mixture of ethyl acetate/hexane as an eluent (10 to 50% EtOAc) to get the desired product HPMA as a white waxy solid (2.4 g, 16.7 mmol, yield 44%).
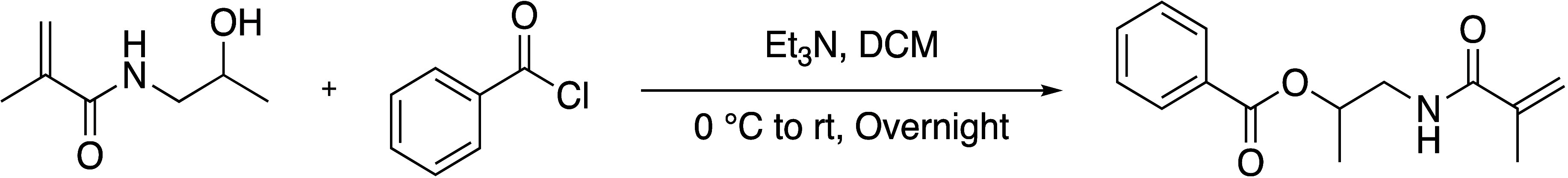


A solution of HPMA (2.4 g, 16.7 mmol, 1.0 equiv.) and triethyl amine (2.8 mL, 20 mmol, 1.2 equiv.) in 20 mL of dichloromethane was maintained at 0 °C under a nitrogen atmosphere. A solution of benzoyl chloride (1.95 mL, 16.7 mmol, 1.0 equiv) in 10 mL of dichloromethane was added dropwise to the reaction mixture with continuous stirring under an inert atmosphere. The reaction mixture was stirred at room temperature overnight. After consumption of the starting material, as judged by TLC analysis, the reaction mixture was filtered through Celite and the solvent was evaporated to get the crude product, which was purified by silica gel column chromatography using a mixture of ethyl acetate/hexane as an eluent (0 to 20% EtOAc) to get the desired product HPMA-Bz as a white waxy solid (2.4 g, 9.7 mmol, yield 58%). ^1^H NMR: (CDCl_3_, 400 MHz), δ (ppm): 8.05–8.02 (m, 2H), 7.60–7.56 (m, 1H), 7.48–7.43 (m, 2H), 6.25 (bs, 1H), 5.66 (brs, 1H), 5.34–5.26 (m, 2H), 3.66 (ddd, *J*_1_ = 14.4 Hz, *J*_2_ = 5.5 Hz, *J*_3_ = 3.5 Hz, 1H), 3.56 (partially merged ddd, *J*_1_ = 14.4 Hz, **J*_*2*_* = 8.4 Hz, *J*_3_ = 5.5 Hz, 1H), 1.93 (bs, 3H), 1.41 (d, *J* = 6.5 Hz, 3H).

### Poly(benzoyloxypropyl methacrylamide) (P(HPMA-Bz)) Synthesis

RAFT polymerization of poly(benzoyloxypropyl methacrylamide) (pHPMA-Bz) was conducted under a nitrogen atmosphere in toluene (40 wt % monomer) at 70 °C for 20 h with 4-Cyano-4-(ethylsulfanylthiocarbonyl) sulfanylvpentanoic acid (ECT) and 2,2′-azobisisobutyronitrile (AIBN) as the RAFT chain transfer agent and initiator, respectively, and the CTA to initiator ratio was 10:1. The reaction mixture solidified during the reaction, and the polymer was solubilized in acetonitrile and 3× precipitated into cold diethyl ether. The purified polymer was then dried in vacuo for 48 h. The polymer was then analyzed by gel permeation chromatography (GPC) to determine its molecular weight and polydispersity (*M*_w_/*M*_n_, PDI) using an EcoSEC Elite GPC System (Tosoh) equipped with a TSKgel Alpha-M column. HPLC-grade DMF containing 0.1 wt % LiBr at 25 °C was used as the mobile phase at a flow rate of 0.6 mL/min. The molecular the polymer was determined using light scattering based on d*n*/d*c* value (0.0105).

### Particle Synthesis

PLGA nanoparticles were formulated using an oil-in-water emulsion method. 100 mg of PLGA Resomer 503 (Sigma), 0–10 mg of P(HPMA-Bz), and 0–10 mg RU.521 or H-151 were dissolved in 1 mL ethyl acetate. Then, the full volume of polymer and drug solution was added dropwise to 2 mL of 4.5% PVA in DI water. The solution was then sonicated using a Sonic Dismembrator (Fisher Scientific Model 120) at 40% amplitude in three 10s increments over ice to keep the solution cold during emulsification. The emulsion was transferred to a round-bottom flask, using 2 mL DI to aid transfer. Ethyl acetate was evaporated using a rotary evaporator at 100 Torr for 45 min to harden the particles. After removing the organic solvent, the solution of particles was washed four times in DI water using 100 kDaA centrifugal filters. Washed samples were transferred to 50 mL conical tubes, flash frozen using liquid nitrogen, and lyophilized to obtain dry nanoparticles. Lyophilized particles were stored at −20 °C for future use.

### Characterization of Nanoparticle Size and Drug Loading

The size of the particles was determined by resuspending dry particles in PBS and performing dynamic light scattering with a Malvern Mastersizer 2000. Drug loading within the particles was determined using HPLC analysis using a Waters e2695 Separations Module (Waters Corporation, Milford, MA) and a Symmetry C18 Column, 100 Å, with 3.5 μm particle size and 3 mm × 150 mm column size. The mobile phase was 50% methanol and 50% 10 mM ammonium acetate in water, and the flow rate was 1.0 mL/min. Absorbance chromatographs were extracted at 252 nm for H-151 and 310 nm for RU.521.

### In Vitro Drug Release

The in vitro drug release of the NP formulations at 37 °C in PBS was determined using Slide-A-Lyzer MINI dialysis devices. To achieve this, a nanoparticle solution containing 300 μg/mL drug was pipetted into the dialysis cup and placed in a 50 mL conical tube containing a PBS sink. These tubes were agitated on an orbital shaker at 37 °C, and 50 μL samples were collected at designated time points. The PBS sink was replaced after 4 h, and again every 24 h. Each collected sample was lyophilized and processed using the HPLC analysis method described above to determine the amount of released RU.521 or H-151 at each time point.

### Fitting Drug Release Data to Models

We fit drug release data to the first order (eq 1), Baker–Lonsdale (eq 2^54^), and Korsmeyer–Peppas (eq 3^55^) models of drug release, shown below.
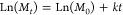
1
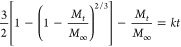
2
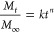
3

In the above equations, *M*_0_ is the initial amount of drug released at time 0, *M*_*t*_ is the amount released at time *t*, *M*_*∞*_ is the total amount of drug in the system, *k* is a kinetic constant for the particular drug/polymer system, and *n* is a release exponent characteristic of the mechanism of drug release.

### Cell Culture

RAW-Dual, THP1-Dual, and THP1-Dual KO-TREX1 cells were purchased from InvivoGen. All cell lines were cultured according to manufacturer’s specifications. Bone marrow-derived macrophages (BMDMs) were isolated from 6- to 8-week-old female C57BL/6 mice and cultured as previously described.^65^ Briefly, bone barrow was flushed from the femurs and tibias of mice using complete BMDM culture medium (RPMI 640 medium supplemented with 10% heat-inactivated FBS, 100 U/mL penicillin, 100 μg/mL streptomycin, 2 mM l-glutamine, 10 mM HEPES, 1 mM sodium pyruvate, 1× nonessential amino acids, and 50 μM β-mercaptoethanol), and the marrow was passed through a 70 μM cell strainer. Cells were centrifuged at 1500 rpm for 5 min, resuspended in ACK lysis buffer (ThermoFisher), and washed with cold PBS. Then, cells were seeded in 100 × 15 mm Petri dishes (Corning Inc.) in complete medium supplemented with 20 ng/mL macrophage colony-stimulating factor (M-CSF). Cells were maintained in a 37 °C incubator supplemented with 5% CO_2_, and culture medium containing M-CSF was replaced on days 3, 5, and 7. On day 8, cells were confirmed to be >90% BMDMs (CD11b^+^F4/80^+^) by flow cytometry.

### Evaluation of Inhibitory Activity in ISG Reporter Cell Lines

RAW-Dual and THP1-Dual cells were plated in white wall clear bottom 96-well plates at a density of 50,000 cells per well and incubated for 24 h. Then cells were cotreated with varying doses of SPINs or relevant control and herring testes (HT DNA) complexed to the commonly used transfection agent Lipofectamine 2000 (L2k). Supernatant was collected 24h after treatment and a Quanti-Luc (Invivogen) assay was used to determine the amount of secreted luciferase following manufacturer’s instructions. Luminescence was quantified using a plate reader (Synergy H1Multi-Mode Microplate Reader; Biotek) using white, opaque-bottom 96-well plates (Greiner Bio-One). The signal for each sample concentration was determined using 3 biological replicates. All reporter cell measurements were normalized by subtracting the average value of a PBS-treated control group. The IC_50_ values were calculated for each of the dose responses using curve fitting analysis in the GraphPad Prism software.

### Cell Viability

Thirty microliters of Cell Titer Glo solution (Promega) was added to each well of treated cells, and the mixture was incubated for 45 min at 37 °C per manufacturer’s instruction. The lysate was then transferred to a white, opaque 96-well plate, and luminescence was read using a plate reader (Synergy H1Multi-Mode Microplate Reader; Biotek) to determine cell viability after treatment.

### Gene Expression in BMDMs

500 000 BMDMs were seeded in a 12-well plate and treated with with HT DNA and SPINs or relevant control for 6h or 24h. Total RNA was isolated using a RNeasy Mini kit (Qiagen, Germantown, MD). Total RNA (1 μg) was reverse transcribed using an iScript cDNA synthesis kit (Bio-Rad) and qPCR was performed using a TaqMan Mastermix kit (Thermo Fisher Scientific) as per manufacturer’s instructions. Primers used included: mouse *Hmbs* (Mm01143545_m1), mouse *Cxcl10* (Mm00445235_m1), mouse *Ifnb1* (Mm00439552_s1), and mouse *Tmem173* (Mm01158117_m1).

### IFN-β Secretion in BMDMs

After 6 or 24 h treatment of BMDMs, the secreted IFN-β concentration in the supernatant was measured with the LumiKine Xpress mIFN-β 2.0 ELISA kit (InvivoGen) according to the manufacturer’s instructions.

### Evaluation of BMDM Activation

BMDM activation was evaluated by flow cytometric analysis of surface CD80, CD86, and CD206 expression. Briefly, 1 × 10^6^ BMDMs/well were seeded in a 12-well plate and treated with HT DNA and SPINs or relevant control for 24 h. The cells were collected and washed with 1% BSA in PBS and then stained with KIRAVIA Blue 520-anti-F4/80 (1:100), APC-anti-CD11b (1:100), PE-anti-CD86 (1:100), and PE-Cy5-anti-CD80 (1:100) (Biolegend) antibodies. Dead cells were excluded from analysis using DAPI (live/dead) stain (1:20 000). Cells were analyzed using a CellStream flow cytometer.

## Supporting Information Available

Viability of cells from experiments represented in Figures 1–3, dose–response curves of cell lines cotreated with empty nanoparticles and HT DNA, SPIN-H efficacy in BMDMs, NP uptake as determined by flow cytometry, and GPC of P(HPMA-Bz) used to make higher-loading SPINs (PDF)
