# Supplementary material for: STING-Pathway Inhibiting Nanoparticles (SPINs) as a Platform for Treatment of Inflammatory Diseases
Source: ACS Appl Bio Mater. 2024 Apr 2;7(8):4867–78. doi: 10.1021/acsabm.3c01305 (PMC11337154; doi:10.1021/acsabm.3c01305)
Supplement: Supplementary file 1 — mt3c01305_si_001.pdf [file mt3c01305_si_001.pdf]

Supporting Information

STING-Pathway Inhibiting Nanoparticles (SPINs) as a Platform for Treatment of Inflammatory Diseases

Lucinda E. Pastora[1], Neeraj S. Namburu[2], Karan Arora[1], Plamen P. Christov[3], and John T. Wilson[1,3-10]*

[1]Department of Chemical and Biomolecular Engineering, Vanderbilt University, Nashville, TN

[2]School for Science and Math at Vanderbilt, Vanderbilt University, Nashville, TN

[3]Vanderbilt Institute of Chemical Biology, Vanderbilt University, Nashville, TN

[4]Department of Biomedical Engineering, Vanderbilt University, Nashville, TN

[5]Department of Pathology, Microbiology, and Immunology, Vanderbilt University Medical Center, Nashville, TN

[6]Vanderbilt Institute of Nanoscale Science and Engineering, Vanderbilt University, Nashville, TN

[7]Vanderbilt Institute for Infection, Immunology, and Inflammation, Vanderbilt University, Nashville, TN

[8]Vanderbilt Center for Immunobiology, Vanderbilt University Medical Center, Nashville TN

[9]Vanderbilt Ingram Cancer Center, Nashville, TN, 37232

[10]Vanderbilt Digestive Diseases Research Center, Vanderbilt University Medical Center, Nashville TN

***To whom correspondence should be addressed:**

John T. Wilson, Ph.D.

2400 Highland Avenue

107 Olin Hall

Nashville, TN 37212

Phone: +1-615-322-6406

e-mail: john.t.wilson@vanderbilt.edu

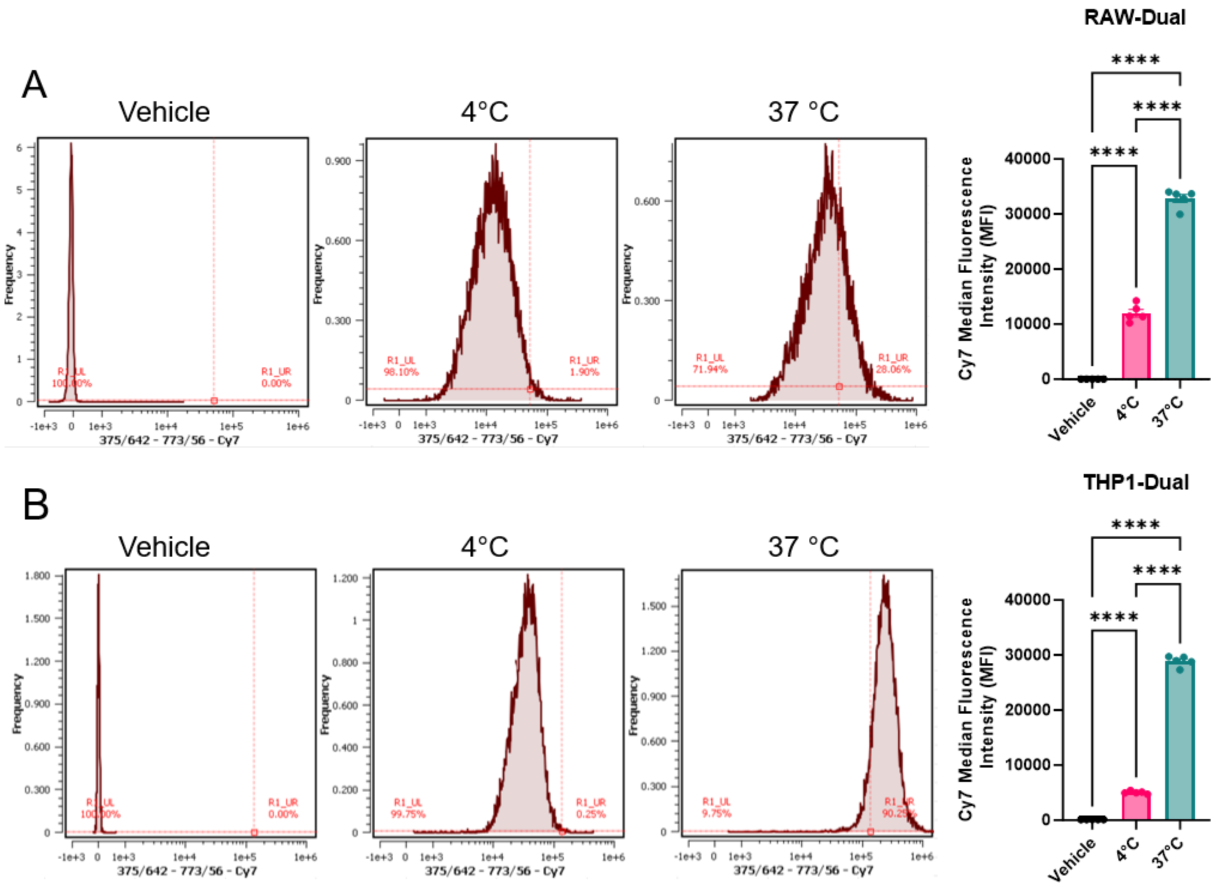

Supplemental Figure 1 – Uptake of Nanoparticles in RAW-Dual and THP1-Dual cells. (A) Example frequency of Cy7 signal in RAW-Dual cells treated with media (Vehicle) or Cyanin7-loaded PLGA NPs, followed by a 6-hour incubation of the treated cells at 4°C or 37°C. The median fluorescence intensity of the cells after incubation was quantified. **(B)** Example frequency of Cy7 signal in THP1-Dual cells treated with media (Vehicle) or Cyanine7-loaded PLGA NPs, followed by a 6-hour incubation of the treated cells at 4°C or 37°C. The median fluorescence intensity of the cells after incubation was quantified. Data is represented as mean ± SEM, n=5. ****P≤0.0001 by one-way ANOVA.

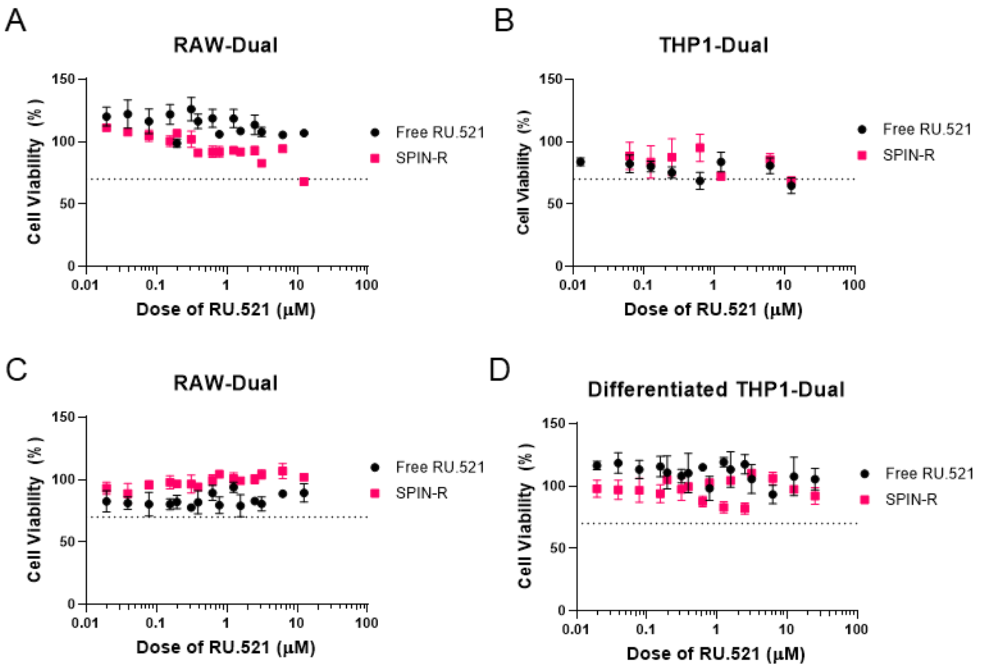

Supplemental Figure 2 – Viability of cells treated with SPIN-R or free RU.521. (A) Viability in RAW-Dual cells, corresponding to data in Figure 1F. **(B)** Viability in THP1-Dual cells, corresponding to data in Figure 1G. **(C)** Viability in RAW-Dual cells after pulse-chase treatment, corresponding to data in Figure 1I. **(D)** Viability in differentiated THP1-Dual cells after pulse-chase treatment, corresponding to data in Figure 1J. Data is represented as mean ± SEM, n=3. Dashed line corresponds to 70% viability threshold.

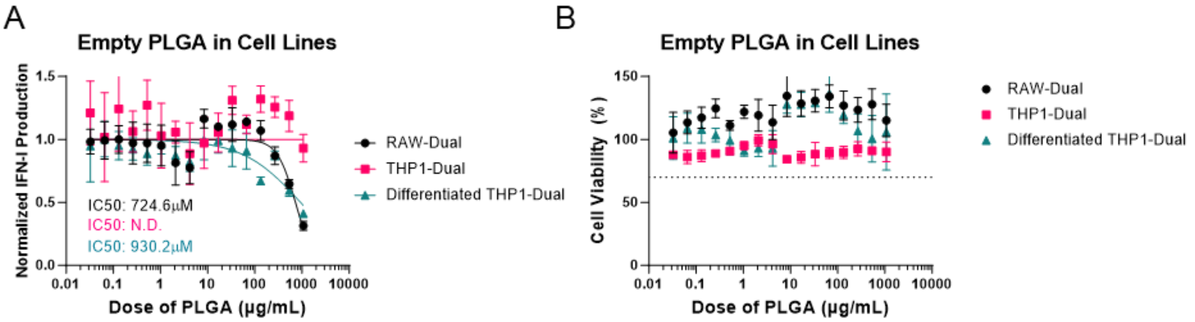

Supplemental Figure 3 – Empty PLGA effect on HT-stimulated cell interferon production and viability. (A) Dose-response curves of IRF3 induced by co-treatment with empty PLGA NPs and 1µg/mL HT DNA in RAW-Dual, THP1-Dual, and differentiated THP1-Dual cells. **(B)** Viability of RAW-Dual, THP1-Dual, and differentiated THP1-Dual cells co-treated with empty PLGA NPs and 1µg/mL HT DNA. Data is represented as mean ± SEM, n=3. Dashed line corresponds to 70% viability threshold.

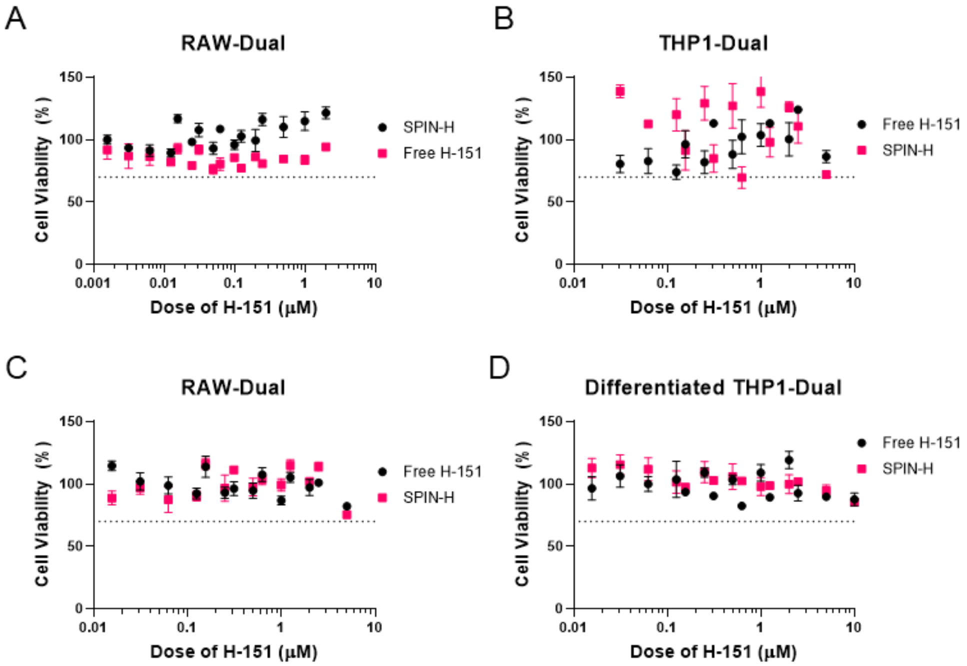

Supplemental Figure 4 – Viability of cells treated with SPIN-H or free H-151. (A) Viability in RAW-Dual cells, corresponding to data in Figure 2E. **(B)** Viability in THP1-Dual cells, corresponding to data in Figure 2F. **(C)** Viability in RAW-Dual cells after pulse-chase treatment, corresponding to data in Figure 2H. **(D)** Viability in differentiated THP1-Dual cells after pulse-chase treatment, corresponding to data in Figure 2I. Data is represented as mean ± SEM, n=3. Dashed line corresponds to 70% viability threshold.

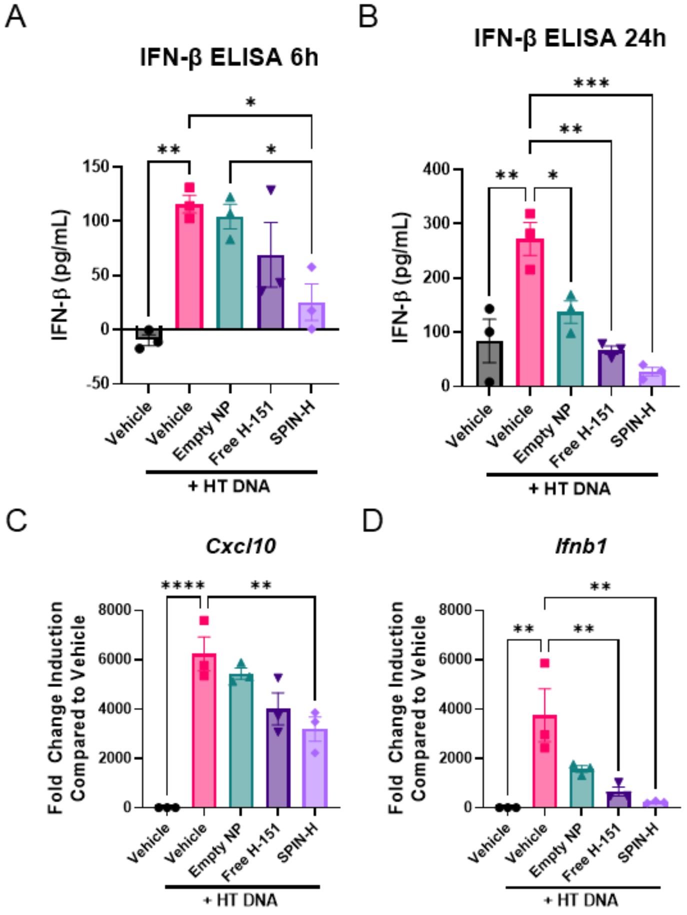

Supplemental Figure 5 – SPIN-H efficacy in BMDMs. A) IFN-β ELISA of supernatants from BMDMs co-treated with the designated SPIN-H or control and 1 μg/mL HT DNA after 6 hours **(B)** and 24 hours (n=3). **(C)** Gene expression of *Cxcl10*, **(D)** *Ifnb1*, and **(E)** *Tmem173* in BMDMs co-treated with the designated SPIN or control and HT DNA for 6 hours (n=3). Data is represented as mean ± SEM. *P≤0.05, **P≤0.01, ***P≤0.001, ****P≤0.0001 by one-way ANOVA.

Analysis Report - Results / Peak 1

Peak-Average Data by Light Scattering			Data by Universal		
Mw	19,173	Da	Mn	0	Da
**Distribution Data by Light Scattering**			Mw	0	Da
Mn	19,413	Da	Mz	0	Da
Mw	20,468	Da	Mp	0	Da
Mz	21,514	Da	Mw/Mn	NaN	
Mp	19,787	Da	**Intrinsic Viscosity**		
Mw/Mn	1.05		Iv,batch	0.00	
**Data By Conventional Calibration**			Iv,w	0.00	
Mn	8,724	Da	**Rg Peak-Average**		
Mw	13,711	Da	Rg,z	11.2	nm
Mz	21,405	Da	**Rg Distribution**		
Mp	11,528	Da	Rg,n	6.8	nm
Mw/Mn	1.57		Rg,w	7.1	nm
			Rg,z	7.4	nm

Distribution Plot

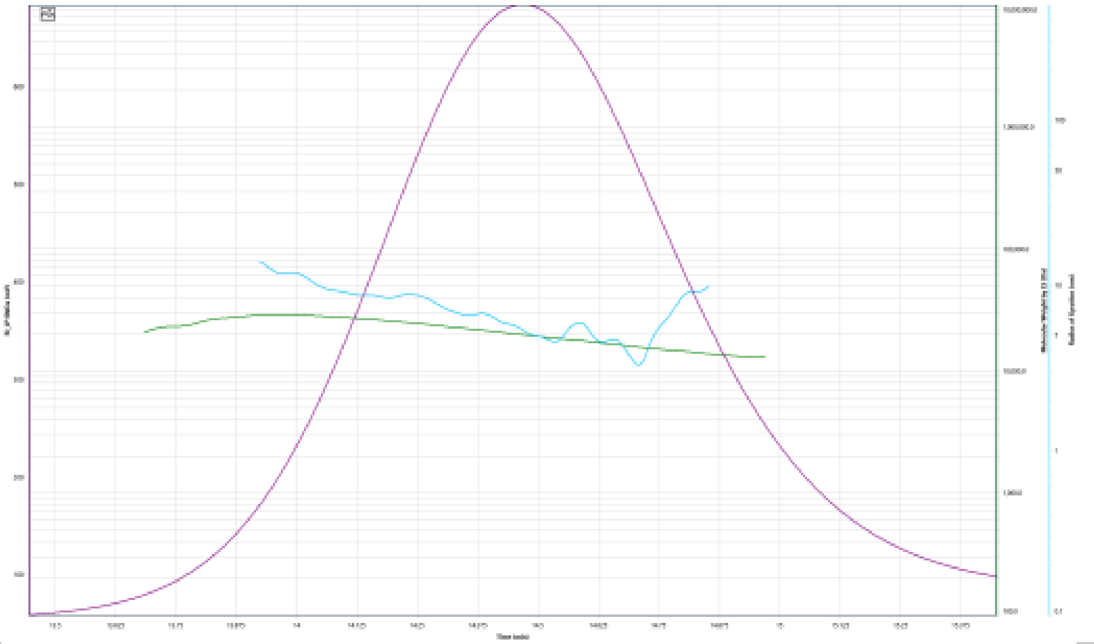

SECview Advanced Multi-Detector SEC Software

12/7/2023

Page 1

Supplemental Figure 6 – P(HPMA-Bz) size and PDI, determined by GPC.

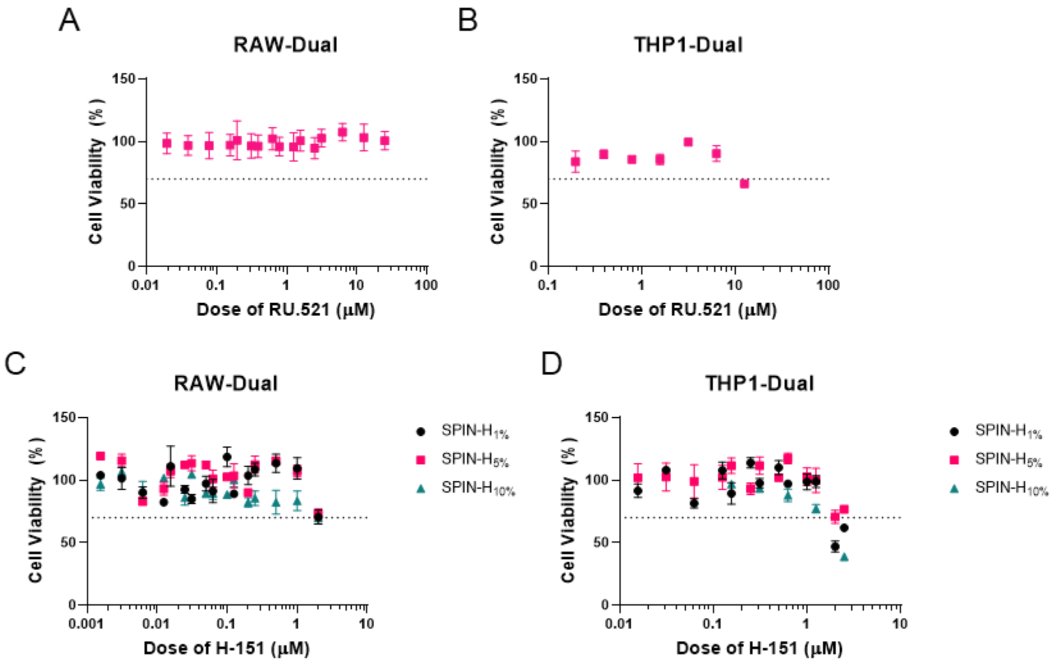

Supplemental Figure 7 – Viability of cells treated with SPINs containing H(HPMA-Bz).
(A) Viability of RAW-Dual cells treated with SPIN-$R_{10\%}$, corresponding to data in Figure 3E.
(B) Viability of THP1-Dual cells treated with SPIN-$R_{10\%}$, corresponding to data in Figure 3F.
(C) Viability of RAW-Dual cells treated with SPIN-$H_{1\text{-}10\%}$ corresponding to data in Figure 3I.
(D) Viability of THP1-Dual cells treated with SPIN-$H_{1\text{-}10\%}$ corresponding to data in Figure 3J.
Data is represented as mean ± SEM, n=3. Dashed line corresponds to 70% viability threshold.
